# Toward Interpretable Cell Image Representation and Abnormality Scoring for Cervical Cancer Screening Using Pap Smears

**DOI:** 10.3390/bioengineering11060567

**Published:** 2024-06-04

**Authors:** Yu Ando, Junghwan Cho, Nora Jee-Young Park, Seokhwan Ko, Hyungsoo Han

**Affiliations:** 1 Department of Biomedical Science, Kyungpook National University, Daegu 41566, Republic of Korea; yuando@knu.ac.kr (Y.A); skanny@knu.ac.kr (S.K.); hshan@knu.ac.kr (H.H.); 2 Clinical Omics Institute, Kyungpook National University, Daegu 41405, Republic of Korea; 3 Department of Pathology, School of Medicine, Kyungpook National University, Daegu 41944, Republic of Korea; pathpjy@knu.ac.kr; 4 Department of Pathology, Kyunpook National University Chilgok Hospital, Daegu 41404, Republic of Korea

**Keywords:** cervical cancer screening, deep learning, variational autoencoders, clustering

## Abstract

Screening is critical for prevention and early detection of cervical cancer but it is time-consuming and laborious. Supervised deep convolutional neural networks have been developed to automate pap smear screening and the results are promising. However, the interest in using only normal samples to train deep neural networks has increased owing to the class imbalance problems and high-labeling costs that are both prevalent in healthcare. In this study, we introduce a method to learn explainable deep cervical cell representations for pap smear cytology images based on one-class classification using variational autoencoders. Findings demonstrate that a score can be calculated for cell abnormality without training models with abnormal samples, and we localize abnormality to interpret our results with a novel metric based on absolute difference in cross-entropy in agglomerative clustering. The best model that discriminates squamous cell carcinoma (SCC) from normals gives 0.908±0.003 area under operating characteristic curve (AUC) and one that discriminates high-grade epithelial lesion (HSIL) 0.920±0.002 AUC. Compared to other clustering methods, our method enhances the V-measure and yields higher homogeneity scores, which more effectively isolate different abnormality regions, aiding in the interpretation of our results. Evaluation using an external dataset shows that our model can discriminate abnormality without the need for additional training of deep models.

## 1. Introduction

Cervical cancer screening is invaluable for the early detection and prevention of cervical cancer [1]. A pap smear is used to screen for cervical cancer by detecting morphological abnormalities [2]. While pap smear screening has been successful at reducing rates of cervical cancer mortality, incidence and mortality have increased in developing countries, owing to the lack of resources [3]. Developing quantitative and autonomous methods using computer technology can potentially improve healthcare worldwide by making the smear screening process cost-efficient.

Despite efforts expended to standardize categories of precancerous cells, interobserver reproducibility of cell classification is still a challenge, owing to the morphological diversity of cells in a pap smear [4,5]. This can lead to underdiagnoses (or overdiagnoses) characterized by false-negative (-positive) rates of the screening method that can negatively affect the health of women. Therefore, a robust and reliable computational approach should encode morphological diversity and score the abnormality of the cell. Recent efforts expended to classify cell abnormalities include the use of hand-crafted features and deep learning. These methods are based on supervised learning; models require fixed labels assuming a gold-standard interpretation that does not consider possible disagreement between observers.

The development of a robust and accurate automated pap smear screening method was motivated by the goal of reducing human errors and examination time [6]. This is achieved by quantitative assessments of either hand-crafted features [7] or the use of deep learning [8,9]. Methods using hand-crafted features are interpretable in the sense that we can calculate the importance or weights of features, unlike deep learning wherein the trained model is usually a black box. A study closely related to ours is that of Özbay et al. [10], where the authors proposed an interpretable deep-learning framework for cervical cancer detection using the “hash” layer. Unlike this method, we can determine the qualitative meaning of each feature dimension by interpreting the images generated by the decoder along a fixed direction in feature space based on the use of the Variational autoencoder (VAE) [11] formulation. This allows comparisons between images by calculating the change in latent space, which quantitatively shows how the images differ.

Most modern image-based deep learning involves a non-linear transformation of an image to a lower dimension space (compared to input), which can also be called image features, to analyze relationships between semantic properties of images [12]. In supervised learning, where the semantic property or label of each image is supplied during training, image features that best discriminate different labels are computed. In contrast, unsupervised learning involves no such additional information on the characteristics of images. Instead, features are learned from image data only and their characteristics are dependent on the inductive biases such as the choice of model and distribution features [13].

VAEs are generative models that assume distribution characteristics on the generating process of data [11]. They are similar to autoencoders [14] in that they both have an encoder that encodes image features to lower dimension and a decoder that recovers the original image using the image features but introduce latent variables that are responsible for the image generation. These latent variables have distribution characteristics, usually a multivariate standard normal distribution, from which we can sample to generate new images. By enforcing a multivariate standard normal distribution (from here on, we call this latent distribution) on the latent variables given training data, images very unlike the training data are expected to have low likelihood with respect to the latent distribution and have latent variables distant from the origin. In this work, we use the terms latent variable, latent vector, and features interchangeably and, by latent space, we mean the space where latent variables are defined.

One-class classification (OCC) [15] using VAEs has the benefit of learning features for the given class in an unsupervised manner. This method has been applied to detect deepfakes by utilizing the reconstruction loss and by comparing the latent features of the input and the reconstructed image [16]. Another study applied the same method to intrusion detection in network flow, but the anomaly score was set to the reconstruction probability [17]. The advantage of our method is that we do not require additional passes to the decoder. We analyze herein only the distribution in the latent space by using the negative likelihood in the feature space as our score. To the best of our knowledge, we are the first to train our encoder using unsupervised learning and the first to apply OCC using VAEs in pap-smear anomaly detection.

Clustering is an unsupervised method used to determine groups of related samples where interpoint distances are small compared with the points outside a cluster [18]. In the context of pap-smear classification, several clustering methods, such as K-Means and its fuzzy variant [19], have demonstrated good performance [20,21]. Unlike previous studies, our approach is unique because we do not rely on fixed points to cluster our samples. Instead, we utilize nonfixed points, mapping the data into a multidimensional latent space represented as a probability distribution. The analysis of data using nonfixed points [22] and calculating statistical distances to compare class distributions has been studied previously [23]. However, to our knowledge, we are the first to apply agglomerative clustering on nonfixed points [24] using an information-theory-based pseudometric. We also demonstrate that, with some additional assumptions, our pseudometric equals the Euclidean distance, which provides evidence for comparable but slightly better results than those obtained using the K-Means with the Euclidean distance.

In this study, we explore an unsupervised method for learning features of normal cells and using negative log-likelihood to quantify abnormality. To interpret our latent space, we analyze the factors of variation learned by our model and introduce a metric based on cross-entropy to cluster similar cells. We test our method against the baselines of other unsupervised clustering algorithms. Our contributions are as follows:

- We demonstrate that VAE models can learn interpretable features from pap-smear datasets;
- The trained models can detect abnormalities by estimating a Gaussian based on the latent feature space using only normal samples;
- Additional image augmentation during the training of generative models can enhance the distinction between normal and abnormal samples;
- The formulation of statistical distance based on cross-entropy enables agglomerative clustering that outperforms conventional clustering methods;
- Finally, our model can be generalized to other datasets containing images of cervical cells. It is capable of distinguishing normal and abnormal images by using the pretrained encoder.

## 2. Materials and Methods

In this section, we define our study’s problem of obtaining an abnormality score function on the latent space of VAEs by first reviewing them in the context of disentanglement. We then define our score function for OCC and follow up using other methods for interpreting latent space by qualitatively assessing reconstructions and using the agglomerative clustering method to obtain similar samples from the input.

### 2.1. VAEs and Disentanglement

Let $S={\left\{X_{i}\right\}}_{i=1}^{N}$ be a random sample of size *N* from an unknown distribution $p_{\theta }$. We assume that *X* is generated by an unobserved continuous random variable *Z*. The set $\left\{p_{\theta }:\theta \in \Theta \right\}$ contains a family of distributions parameterized by θ in Θ and the true but unknown parameter θ.

Owing to the intractability of marginal likelihood computation $p_{\theta }\left(x\right)$ and computation costs occurring from large datasets, Kingma et al. [11] proposed the autoencoding variational Bayes method, in which a recognition model $q_{\phi }\left(z|x\right)$ is approximated to the posterior model $p_{\theta }\left(z|x\right)$. This allows efficient approximation of maximum likelihood or maximum a posteriori approximation for θ, posterior inference of the latent variable *z* given an observed value *x* given θ, and marginal inference of the variable *x*. The recognition model $q_{\phi }\left(z|x\right)$ can be considered as a probabilistic encoder that models the distribution of latent representation z. Likewise, the generative model $p_{\theta }\left(x|z\right)$ can be considered as a probabilistic decoder that models the distribution of the generated datapoint *x* with respect to a latent variable *z*.

We denote $\theta_{d}$ as the parameters for the decoder, $\theta_{e}$ as the parameters for the encoder, $q_{\theta }$ as the approximate posterior parameterized as a diagonal multivariate normal distribution, and $p_{\theta_{g}}$ as the true data-generating distribution with marginal latent distribution as a standard normal distribution. The evidence lower bound (ELBO) of VAE [11] is

(1) $$L\left(\theta_{d},\theta_{e}\right)=L_{r}\left(\theta_{d},\theta_{e}\right)-\frac{1}{\left|S\right|}\sum_{x\in S}D_{KL}(q_{\phi }\left(z|x\right)\left|\right|p_{\theta }\left(z\right)),$$

where $D_{KL}$ is the Kullback–Leibler divergence [25]. Burgess et al. [26] showed that, for curated datasets, adding an extra term ß to the ELBO helps disentangle factors of variation [27], which may help interpret features. The ELBO for β-VAE is as follows:

(2) $$L\left(\theta_{d},\theta_{e};\right)=L_{r}\left(\theta_{d},\theta_{e}\right)-\frac{1}{\left|S\right|}\sum_{x\in S}\left[\beta D_{KL}(q_{\theta_{d}}\left(z|x\right)||p_{\theta_{g}}\left(z\right))\right].$$

Chen et al. [28] showed that total correlation [29], a term found by decomposing ELBO [30], is related to the disentangling property found in ß-VAE. The ELBO for their model, ß-TCVAE is as follows:

(3) $$L\left(\theta_{d},\theta_{e};\alpha ,\beta ,\gamma \right)=L_{r}\left(\theta_{d},\theta_{e}\right)-\alpha I_{q}\left(z;x\right)-\beta D_{KL}(q\left(z\right)\left|\right|\prod_{j}q\left(z_{j}\right))-\gamma \sum_{j}D_{KL}(q\left(z_{j}\right)||p\left(z_{j}\right)),$$

where *I* is the mutual information function and α=1=γ were the values of constants for all experiments in this study.

In this study, we explored the possibility of interpreting latent factors for cells in pap-smear images using different loss functions of VAE. By performing training using only normal samples, we hypothesized that color and morphological factors may be found in the latent space so that we can interpret results when inputs show signs of abnormality.

### 2.2. Score Function

The ELBO suggests that our approximate posterior $q_{{\hat{\theta }}_{e}}\left(z|x\right)$ should be a factorized standard normal distribution after training. However, our distribution of normal samples may differ from training samples, owing to augmentations applied during training. Instead, we also estimated our distribution of normal samples by a multivariate normal distribution. We define our scoring function *s* by the negative log-likelihood of a multivariate normal distribution:

(4) $$s\left(z\right)=-\log N\left(z|x,\hat{\mu },\hat{V}\right),$$

where $\hat{\mu }$ and $\hat{V}$ are the estimated parameters from normal cell image examples.

### 2.3. Interpreting Latent Space

The formulation of VAEs suggests independent image-generating factors in latent space. By traversing along the latent entry, we can infer qualitatively the meaning of each factor in latent space.

As we modeled the image distribution as an augmented distribution, we traversed along each entry centered at the mean of the nonaugmented data distribution for 10 steps, starting from a value equal to −4 × standard deviations (with respect to the mean) to +4 × standard deviations (with respect to the mean).

Another way we can interpret latent space is to identify samples near the encoded input. The straightforward approach is to cluster samples in latent space and identify other samples in the cluster associated with the input. However, a characteristic of VAEs may allow us to use statistical distances instead of sampling approaches to this problem. The reparameterization trick [11] for a Gaussian posterior, a differentiable transformation $g_{\theta_{e}}$, yields

(5) $$z=g_{\theta_{e}}\left(x,\epsilon \right)=\mu \left(x;\phi_{\mu }\right)+\sigma \left(x,\phi_{V}\right)\epsilon$$

where $\phi_{\mu }$ and $\phi_{V}$ are parameters for the mean and standard deviation for the latent feature *z*. This reveals that the latent feature vector z is modeled by μ with an error term with variance $\sigma^{2}$. Therefore, instead of using contemporary clustering methods that use the mean vector or a sample from this distribution, we can use statistical distances. Images in the same cluster should contain similar image characteristics, owing to the proximity of their locations in latent space. By incorporating statistical distances, we hypothesized that our method clusters similar images more effectively compared with contemporary methods, such as K-means with Euclidean distance, by utilizing the error of representation and factoring relative information of nearby samples.

### 2.4. Cross-Entropy-Based Referenced Statistical Distance

We introduce a statistical distance that is related to well-known divergences, such as the Jenson–Shannon and Jefferys, and an equality to Mahalanobis distance as a special case, as indicated in the this section. We call this cross-entropy-based referenced statistical distance (CRSD). Connnections of CRSD to other divergences are descriped in Appendix A.

Given two probability distributions *p* and *q*, the CRSD with respect to a probability distribution *r* is defined as

(6) $$d_{CRSD}\left(p,q;r\right)=\left|CE\left(r,q\right)-CE\left(r,p\right)\right|,$$

where the cross-entropy of *q* relative to *p* is defined by CE(p,q)=∫p(x)logq(x)dx. Henceforth, for brevity purposes, we eliminate the acronym. Based on this definition, it can be inferred that the function is symmetric, d(p,q;r)=d(q,p;r), non-negative, and zero if p=q. However, d(p,q;r)=0 does not imply that p=q, and this can easily be demonstrated by Gaussian distributions with centered *r* values; *p* and *q* have means equal to μ and −μ. The triangular inequality is also satisfied and proven using simple algebra. We also define for multiple reference probability densities: if *R* is a set of probability densities, the CRSD with respect to *R* is defined by

(7) $$d\left(p,q;R\right)=\sum_{r\in R}\left|CE\left(r,q\right)-CE\left(r,p\right)\right|$$

In this study, we substituted the Euclidean distance for CRSD in the agglomerative clustering algorithm [24] when we used nonfixed points. We restricted *R* to be finite in our experiments to define the latent distributions in a given piece of data related to training, validation, or test fold.

### 2.5. Dataset

A publicly available conventional pap-smear dataset provided by the CRIC platform [31] was chosen for our experiment. This dataset contained 400 pap-smear images and 11,534 classified cells, which contained more classified cells than other open datasets [32,33]. The dataset contained six classes according to the Bethesda system [4], namely, NILM, LSIL, HSIL, ASC-US, ASC-H, and SCC. As the classified cells were annotated rather than cropped, different views of cells (achieved by rotation and displacement) could be obtained during training without introducing background elements generated by augmentation on cropped images. In addition, we removed some annotations that were close to the boundary of the image (within 128 pixels with respect to either the x- or y-axis). The numbers of cropped images used in our experiment are shown in Table 1.

To check for the generalizability of our model, we used another publicly available SIPAKMED [33] dataset to compare scores between abnormal and normal cells. The SIPAKMED dataset contained information on cell morphological information, which we used to determine the cell coordinates. The dimension of all cell images was 256 × 256; these images were center-cropped to 64 × 64 before they were input into the model. This limited the field of view of our model and allowed focus on the nucleus of a single cell and its immediate surroundings.

### 2.6. Experiments

**Figure 1:**
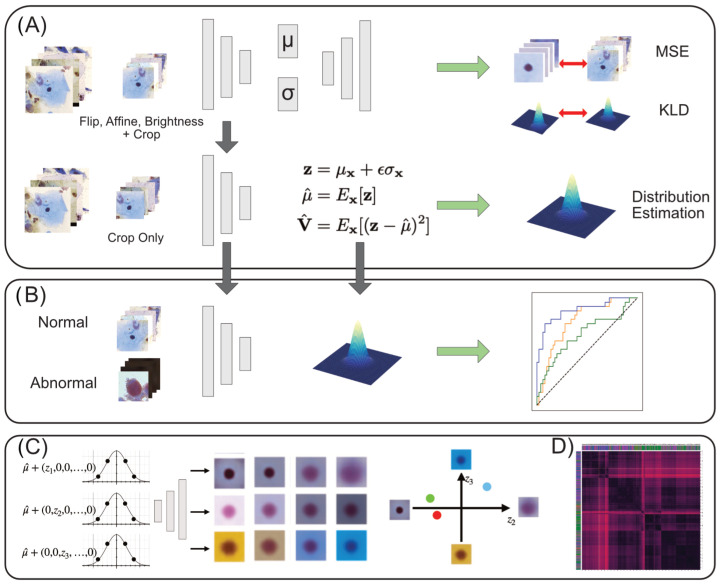
Overview. (**A**) The process of training VAEs and estimation of the latent normal distribution. See Appendix B for details on the model architecture. (**B**) Using the estimated normal latent distribution, we compare likelihoods between normal and abnormal latent vectors to calculate ROC curves for performance evaluation. (**C**) The latent space is traversed to assess the morphological meaning of each dimension, after which the latent feature can be interpreted quantitatively for each image. (**D**) Clustering is performed to analyze the local characteristics in latent space and to obtain similar image examples.

Our study comprises four parts: training of VAEs and the estimation of normal sample distribution in latent space, abnormality scoring, interpretation method for latent representations, and clustering. An overview is shown in Figure 1.

We trained VAE, β -VAE, and β -TCVAE using normal samples (NILM) in the CRIC dataset to learn disentangled features and to calculate a simple scoring function to discern abnormal samples. Our implementation was based on Subramanian [34]. Five-fold cross-validation was performed on a two-class classification task (NILM vs. abnormal) to identify the best hyperparameters. The hyperparameters were varied using β = 1, 4, 16, and the latent dimensions 8, 32, and 128.

Additionally, image-augmentation techniques were varied to determine if additional image preprocessing had a positive effect on model performance. In generative models, severe augmentations tend to distort the learned data distribution; thus, we selected plausible augmentations, such as rotation, translation, and brightness adjustment, that mimic possible variations in the usual data acquisition process using an optical microscope. Three methods were tested as hyperparameters in the cross-validation process: none; random flip, rotation, and translation (FA); and random flip, rotation, translation, and brightness (FAB). For each abnormal class, scores were then compared against NILM to assess the per-class performance of our model. Finally, clustering algorithms were compared to our best model. Clustering algorithms included agglomerative clustering [24], K-means [35], spectral clustering [36], and DBSCAN [37]. We chose homogeneity, completeness, and V-measure [38] as metrics for comparison. We conducted tests using 100 clusters, and other parameters were set to default using sci-kit learn implementation [39].

We experimented on an additional external dataset, SIPAKMED, to see if our model could score abnormality with comparable or better performance. The model was fixed to only encode images to latent space. The distribution of normal images was estimated using the same procedure as that used for the CRIC dataset. We estimated the Gaussian using different folds of normal data (5-fold split on training datasets). Abnormal data were split into 5 folds, and each sub-dataset was evaluated against normal data to calculate the ROC curve.

## 3. Results

### 3.1. Latent Space Captures Morphological and Color Characteristics of Cervical Pap-Smear Cells

**Figure 2:**
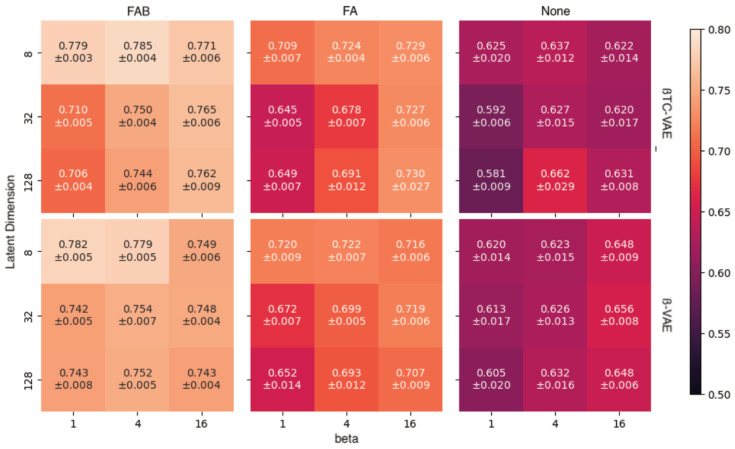
Area under receiver operating characteristic curve (AUROC) for combinations of data augmentation and hyperparameters (ß and latent dimension).

In supervised learning, incorporating additional augmentations tends to enhance the performance of deep models, but this may not directly translate to generative models (such as VAEs) because augmented samples distort the target data distribution that is required to be learned. Therefore, our experiments were conducted on relatively plausible augmentations: rotation of cells, systematic error in centering a cell in an image, and brightness configuration, which depends on the light source of the microscope. Three augmentation methods were tested: no augmentation (None), rotation and translation (FA, flip, and affine), and FA with brightness adjustment (FAB, flip, affine, and brightness). Figure 2 shows that, for any hyperparametric combination, using the full augmentation (FAB) yields the best area under the receiver operating characteristic curve (AUROC) on average. The best model was found to be β-TCVAE with β = 4 and with a latent dimension equal to eight. Further performance analyses were performed using this model.

**Figure 3:**
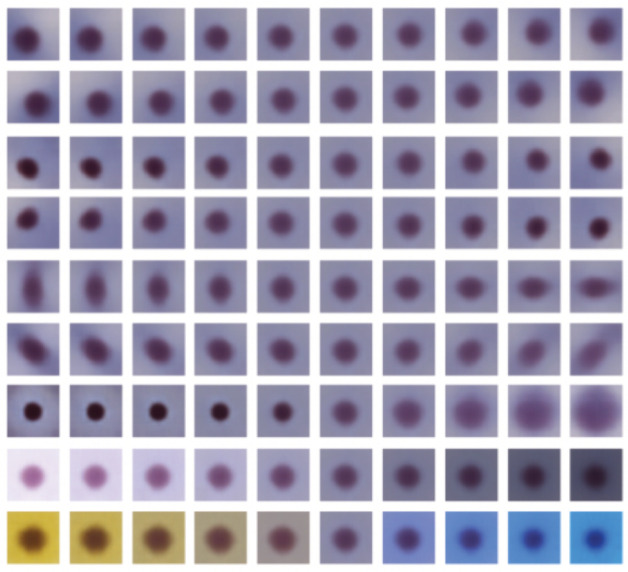
Latent traversals across different dimensions of the ß-variational autoencoder (VAE) (ß = 4 and latent dimension = 32).

Morphological and color-related features were learned by the model using unsupervised training. Figure 3 shows features learned by the ß-VAE model with ß = 4 and 32 latent dimensions. The reconstructed images were obtained by calculating the mean of latent features of normal samples and traversing from −4 to 4 standard deviations from the mean in 10 steps. The top seven rows correspond to the morphology of the cell image concerning the locations, shapes, and sizes of the nuclei. The bottom two rows correspond to color. Images in the second row from the bottom correspond to brightness adjustment, and images in the last row correspond to hue adjustment from gold-brownish to blue.

**Figure 4:**
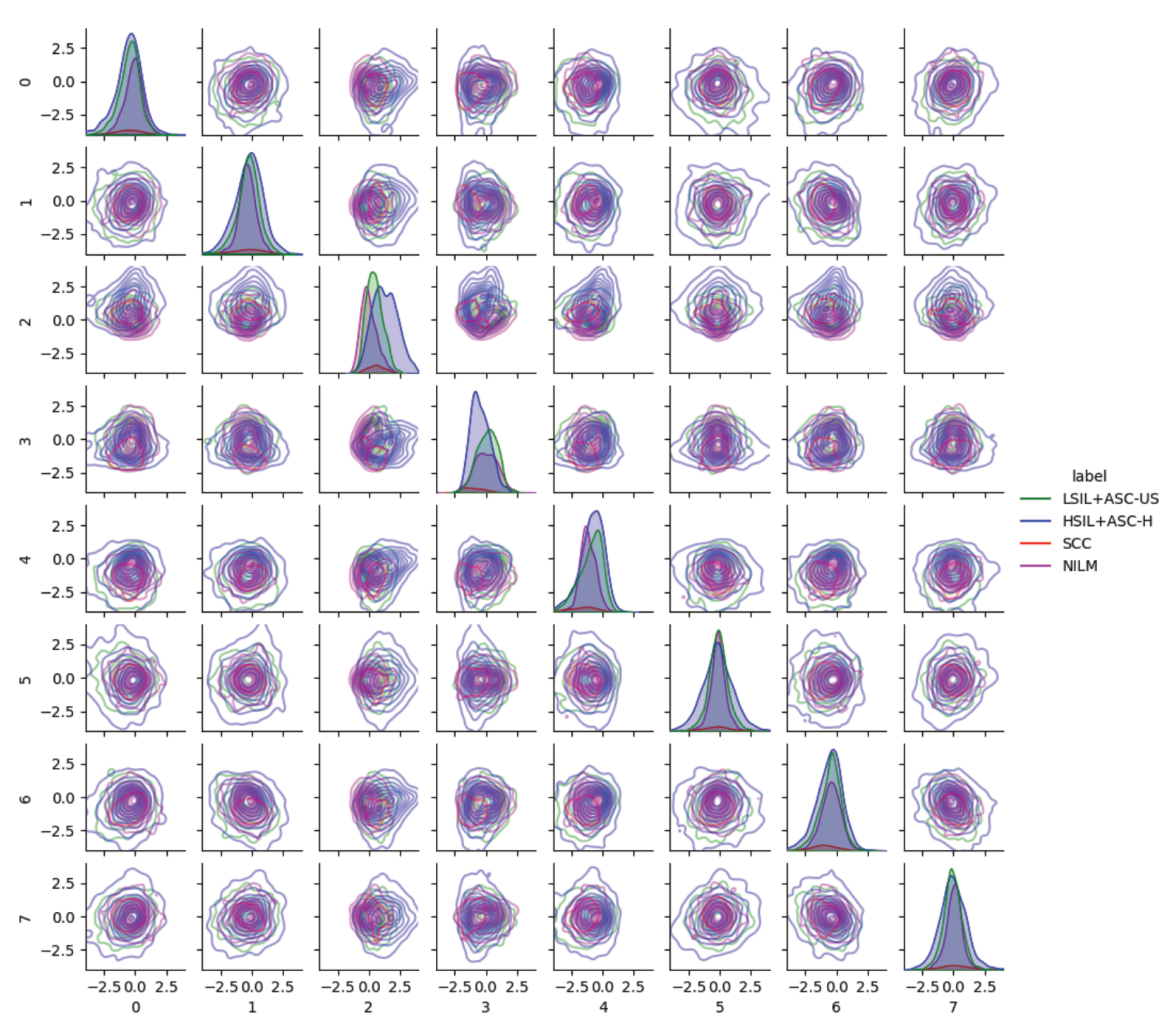
Pairwise latent distribution estimation plots obtained using latent vectors of different classes in the Center for Recognition and Inspection of Cells (CRIC) dataset in conjunction with the use of the best model.

Pairwise latent distribution contour plots between latent dimensions of the best model (ß-TCVAE, ß = 4, and a latent dimension equal to 8) were drawn for the Center for Recognition and Inspection of Cells (CRIC) dataset. Figure 4 shows that each abnormal class contains different image characteristics, even though the encoder/decoder model was trained only based on normal examples, namely, the negative intraepithelial lesion or malignancy (NILM). Each row from top to bottom (left to right columns) corresponds to the latent dimension index (ranging from zero to seven). The following abnormal classes were merged for clarity in the pairwise KDE plots: low-grade intraepithelial lesion (LSIL) with atypical squamous cell with undetermined significance, and high-grade intraepithelial lesion (HSIL) with atypical squamous cell—cannot exclude HSIL (ASC-H). As aforementioned, we observed a shift in the data distribution from the standard normal for NILM in latent space. Scoring based on the NILM latent distribution should account for this shift instead of relying on the formulation of the latent distribution as a standard normal according to the VAE formulation.

### 3.2. Negative Log-Likelihood Is in Line with the Progressive Severity of Precancerous Stages

As atypical cells should be different from normal cells, a score was devised based on negative log-likelihood to quantify the degree of departure from a fixed set of normal cells. NILM latent normal distribution parameters were estimated separately from NILM images that were obtained from model training. We calculated the performances of our scoring function by comparing each abnormal class against normal samples. Table 2 lists accuracy, AUC, F1 scores, and sensitivity and specificity outcomes for each abnormal class. The model performed well at discerning squamous cell carcinoma (SCC) and HSIL from normal cell states followed by the ASC-H, LSIL, and atypical squamous cell of undetermined significance (ASC-US). The AUC scores correspond to the severity of the lesion; AUC scores are ordered from high grades (HSIL, SCC, and ASC-H) to low grades (LSIL and ASC-US).

**Figure 5:**
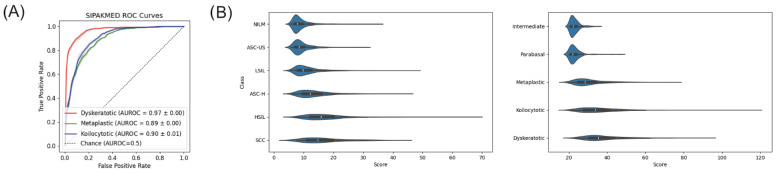
(**A**) Receiver operating characteristic (ROC) curves for each abnormality using external dataset SIPAKMED. (**B**) Violin plots for the CRIC (left), SIPAKMED (right) datasets.

We tested for generalizability by estimating and evaluating the score function for additional datasets using our model. Figure 5A shows the receiver operating characteristic (ROC) curves of each abnormal class for each external dataset SIPAKMED [33]. On average, over cross-validation folds, the AUC values for dyskeratotic, metaplastic, and koilocytotic cells were 0.97, 0.89, and 0.9, respectively.

Figure 5B shows violin plots of scores for each class in each dataset to visualize distribution differences between normal and abnormal cases. Our best model characterizes abnormality as a departure from normality as seen by an increase in the median score with a very long tail response. This pattern is consistent with the other external datasets. However, long tails of normal cells within each dataset indicate irregularity even in normal cases, thus suggesting that score alone may not be a robust classification metric for cells.

### 3.3. Characteristics of External Datasets Revealed by Distributions in Latent Space

**Figure 6:**
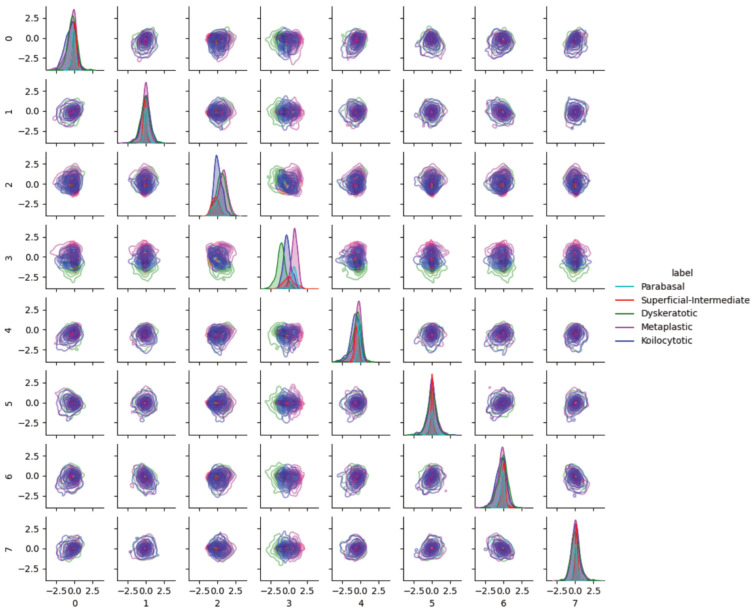
Pairwise latent distribution estimation plots obtained using latent vectors of different classes in the SIPAKMED dataset and the best model.

In addition to pairwise KDE visualization of the CRIC dataset, we analyzed the latent space for possible batch effects. Figure 6 show the pairwise KDE plots with the best model of the latent distributions of SIPAKMED. One observation regarding latent feature index 3 is the differences of modes among abnormal classes in SIPAKMED. We found that feature 3 was associated with color and, in turn, associated with koilocytotic, dyskeratotic, and metaplastic cells in the SIPAKMED data (orange, bluish, and lighter bluish/green). Rote interpretation based on the latent feature value is difficult because feature values are conditioned on the training data (color distribution of normal cell images in the CRIC dataset); therefore, generalization is not guaranteed. However, this strongly suggests the need for further study on batch correction methods within the latent space.

### 3.4. Novel Statistical Pseudo-Distance Improves Clustering Performance

Recognizing that the latent space corresponds to varying morphological and color features, images with similar qualitative features should exist in latent space. Therefore, we applied clustering algorithms to localize similar images. Homogeneity, completeness, and V-measure [38] were calculated for each clustering algorithm to compare performances. Furthermore, as there is uncertainty associated with latent features, we developed a pseudometric known as cross-entropy-based statistical divergence (CRSD) for use in conjunction with conventional clustering algorithms. Cross-validation (fivefold) on the abnormal dataset was performed to compare different unsupervised algorithms using the best model. Table 3 shows that agglomerative methods yield higher V-measures than other methods with agglomerative clustering with CRSD (Agg-SM) as the best-performing algorithm. Homogeneity for agglomerative clustering with Euclidean distance (Agg-EM) and K-Means were similar, but K-Means yielded lower V-measures. Spectral clustering and density-based spatial clustering of applications with noise (DBSCAN) yielded very low V-measures compared with other algorithms, but DBSCAN had the highest completeness score, suggesting that the algorithm favors a smaller number of clusters.

### 3.5. Toward Reduced Intraobserver Variability Using Retrieved Images

**Figure 7:**
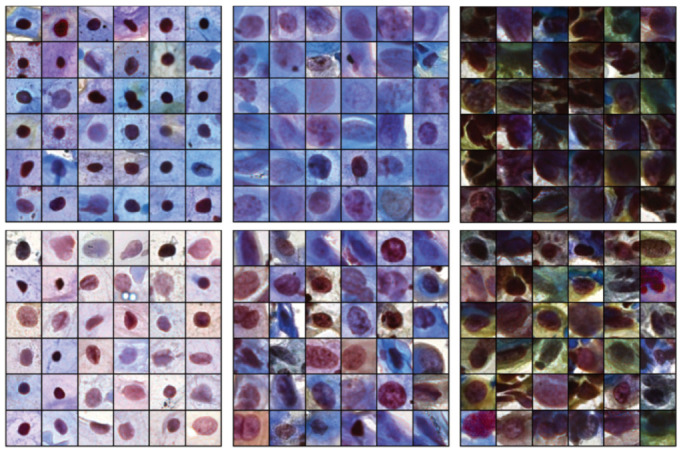
Queried images from two high-homogeneity clusters for each class: negative intraepithelial lesion or malignancy (NILM) (left), low-grade intraepithelial lesion (LSIL) + atypical squamous cell of undetermined significance (ASC-US) (middle), and high-grade intraepithelial lesion (HSIL) + ASC—cannot exclude HSIL (ASC-H) (right).

Related images can be assessed to inspect images within the same cluster. Figure 7 shows two different clusters (top row and bottom) with high NILM homogeneity (first column), LSIL + ASC-US (second column), and HSIL + ASC-H (third column) in the CRIC dataset. Images with similar hue, nuclear shape, and density values are close to each other, demonstrating some consistency with the semantic meaning of latent features. The disadvantage of this approach is the fact that we cannot obtain a cluster with a high homogeneity score in cases in which certain classes have very low image counts within the dataset (such as SCC). This methodology could be improved with more cell images and additional cell labels by multiple pathologists.

## 4. Discussion

VAEs enable the disentanglement of latent features, which allows modeling of the latent space of cervical cells in pap-smear images. Experiments showed that we were successful in obtaining features corresponding to cytoplasmic color, nuclear shape, and image brightness in an unsupervised manner and concurrently achieved high AUROC for SCC and HSIL. More importantly, a simple Gaussian estimation of the latent feature space of normal examples produced these results without additional parameterized model-training requirements. Adding additional augmentations during the training of a generative model improved the discerning capability. However, as the generative model learns the data distribution, applying image augmentation can distort the original data distribution [40]. This is also shown in Figure 4, where the normal class (NILM) is not represented by a centered normal distribution for each latent entry. We deduce that the high discerning performance, despite the shift in latent distribution of NILM images, is due to the use of the estimate normal distribution rather than a standard normal distribution, as induced by the VAE training objective. A comparison of our results with those of other studies showed that there is a trade-off in using a limited number of labeled samples for deep feature learning. Table 4 lists the performance outcomes of other models obtained with supervised training. Despite training using only normal examples, our model was able to discern abnormality with an accuracy of 0.7 in binary classification tasks. This may be due to the low AUC scores for low-grade lesions (LSIL and ASC-US), as indicated in Table 2, which contributes to the overall lower performance.

The choice of evaluation metric for the clustering algorithm was based on the qualitative assessment of the latent space. Each class should be ideally situated in a single cluster for direct interpretation, i.e., ad hoc labeling of the cells within the cluster. However, as our latent space contains features related to position and affine transformation, abnormal classes are not characterized locally in latent space but form local clusters in some dimensions (for example, color and nuclei radii) and deviations from others (position). This suggested the use of separate metrics to quantify clusters containing only a single class; given a specific class, these quantify the capability of performing clustering using a single cluster instead of relying on a single metric such as accuracy. As expected, clustering results show that classes are locally close in the latent space, owing to the higher homogeneity and lower completeness scores, which indicate the sparsity of clusters within this space. This highlights the necessity of querying nearby images to assess new cells when applied in practice. This study was associated with some limitations. While our model can detect abnormalities for most classes, it does not discriminate effectively among abnormal classes like supervised models. To overcome this, we devised a querying method using agglomerative clustering. Images contained in some single cluster should have similar features.

## 5. Conclusions

VAEs provide means to interpret latent features by examining the reconstructed output and give a simple formulation for scoring abnormality. OCC of normal cells in pap-smear images can predict cell abnormality without using abnormal labels. Performance was shown to increase when additional image augmentation was applied during training. Along with interpreting latent features directly, a cross-entropy-based pseudo-metric was introduced for agglomerative clustering. The method consistently outperformed other common unsupervised clustering algorithms. We have shown that, although binary classification performance is worse than the performances of supervised methods, using OCC can discern HSIL and SCC from normal with high accuracy. We also demonstrated an implementation of an interpretable method that may contribute to the development of explainable artificial intelligence (AI) and AI trust in healthcare.

## Figures and Tables

**Table 1:** Number of samples per class from each dataset used in this study.

Dataset	Class	Counts (Total)
CRIC	Negative intraepithelial lesion or malignancy (NILM)	5422
	ASC-US	563
	LSIL	1287
	ASC-H	894
	HSIL	1609
	SCC	156
SIPAKMED	Superficial–Intermediate	831
	Parabasal	782
	Koilocytotic	814
	Dyskeratotic	794
	Metaplastic	785

**Table 2:** Class-wise metrics for the best model. We calculated metrics for each abnormal class compared with those based on the normal dataset. The model was good at discerning squamous cell carcinoma (SCC) and high-grade intraepithelial lesion (HSIL) from normal followed by atypical squamous cell—cannot exclude HSIL (ASC-H), low-grade squamous intraepithelial lesion (LSIL), and atypical squamous cell with undetermined significance (ASC-US).

Abnormal Class	Accuracy (±Standard Deviation (std))	Area under Receiver Operating Characteristic Curve (AUROC) (±std)	F1 Score (±std)	Sensitivity (±std)	Specificity (±std)
SCC	0.806 ± 0.029	0.908 ± 0.003	0.883 ± 0.020	0.800 ± 0.035	0.870 ± 0.036
HSIL	0.849 ± 0.002	0.920 ± 0.002	0.850 ± 0.006	0.829 ± 0.027	0.871 ± 0.026
ASC-H	0.742 ± 0.005	0.808 ± 0.006	0.787 ± 0.007	0.731 ± 0.018	0.761 ± 0.020
LSIL	0.647 ± 0.006	0.689 ± 0.005	0.684 ± 0.018	0.672 ± 0.044	0.613 ± 0.043
ASC-US	0.449 ± 0.030	0.549 ± 0.013	0.492 ± 0.055	0.358 ± 0.058	0.724 ± 0.055

**Table 3:** Unsupervised clustering algorithm comparisons. Homogeneity (h), completeness (c), and V-measure (V) are calculated for each algorithm with number of clusters used. Values close to one indicate better performance. Our method, agglomerative clustering with CRSD (Agg-SM), yields the best V-measure, followed by K-Means. Homogeneity was similar between agglomerative clustering using Euclidean distance (Agg-EM) and K-Means.

Algorithm	Homogeneity (h)	Completeness (c)	V-Measure (V)
Agg-SM	0.393 ± 0.0117	0.151 ± 0.00259	0.218 ± 0.005
Agg-EM	0.282 ± 0.00591	0.0940 ± 0.00174	0.141 ± 0.00265
K-Means	0.275 ± 0.006	0.725 ± 0.0019	0.114 ± 0.003
Spectral clustering	0.0526 ± 0.0031	0.0950 ± 0.0044	0.0676 ± 0.0025
DBSCAN	0.000234 ± 0.000413	0.746 ± 0.414	0.000463 ± 0.000818

**Table 4:** Binary classification results. Supervised learning outperforms one-class classification (OCC) with variational autoencoders (VAEs).

Model	Sensitivity	Specificity	Accuracy	F1	Method
Diniz et al. (Random Forest)	0.958	0.958	0.958	0.958	Supervised
Diniz et al. [41] (Ensemble)	0.96	0.96	0.96	0.96	Supervised
CVM-Cervix [42]	0.916	Not Available	0.9172	0.852	Supervised
Ours (ß- TCVAE with FAB)	0.759	0.683	0.70	0.58	OCC

## Data Availability

Center for Recognition and Inspection of Cells (CRIC) Searchable Image Dataset can be obtained at https://database.cric.com.br (accessed on 1 January 2023). SIPAKMED dataset can be obtained at https://www.cs.uoi.gr/~marina/sipakmed.html (accessed on 1 January 2023).

## Appendix A. Relationships among CRSD and Other Divergences

The distance is equal to the Kullback–Leibler, Jefferys, and Jenson–Shannon divergences, depending on which reference probability distribution or set of probabilities is chosen. We denote the divergences as $D_{KL}$, $D_{J}$, and $D_{JSD}$, respectively. The following are some relations of our formulation to well-known divergences [25]:

(A1) $$d(p,q;p)=D_{KL}\left(p|q\right)$$

(A2) $$d(p,q;\{p,q\})=2D_{J}\left(p,q\right)$$

(A3) $$d(p,q;\{p,q\})\geq 4D_{JSD}\left(p,q\right)$$

If *p* and *q*, are Gaussian distributions with equal covariances, we can express the squared Mahalonobis distance as

(A4) $$d\left(p,q;\left\{p,q\right\}\right)={\left(\mu_{p}-\mu_{q}\right)}^{T}\Sigma^{-1}\left(\mu_{p}-\mu_{q}\right).$$

Additionally, if Σ=I, where *I* is an identity matrix, we are then essentially calculating distances between latent distributions by the Euclidean norm between the means, i.e., $d\left(p,q;\left\{p,q\right\}\right)={∥\mu_{p}-\mu_{q}∥}_{2}^{2}$. Therefore, a connection is made to our method using standard clustering algorithms and the mean vectors of the latent representations using statistical distances; the former is a special case of the latter based on assumptions on the covariances and the choice of the reference distributions.

## Appendix B. Model Architecture, Training Details, and Hardware

Convolutional neural network models used in this work all had the same architecture except for the projection of the encoder/decoder output to latent space. The encoder for the VAEs consisted of 5 convolutional layers with 3 × 3 kernels, 1 padding, and a stride of 2, followed by a 2-dimensional batch normalization [43] and a leaky ReLU non-linear activation [44]. For each layer, channels were 32, 64, 128, 256, and 512, respectively. The feature output of the final convolution layer is flattened, followed by a fully connected layer reducing the dimension to 512 before projecting to latent dimensions that parameterize the mean and log standard deviation by another fully connected layer each. The decoder consisted of 5 transposed convolutional layers with 3 × 3 kernels, 1 padding, 1 output padding, and a stride of 2, followed by a 2-dimensional batch normalization and a leaky ReLU non-linear activation. Before decoding, the sampled latent vectors were projected to the same size as the output of the last layer of the encoder using a fully connected layer. The optimizer used was Adam [45] with a learning rate of 1 × 10^−3^ and with weight decay 1 × 10^−5^. Cosine annealing was applied with $T_{\max}$ equal to 800 using the pytorch implementation [46]. Training was set to 4000 epochs with gradient clip value 15.0. Floating point precision was used. Models were trained in parallel during hyperparameter tuning across three Nvidia RTX A6000 graphics processing units and each training session took about 33 h.
